# A Chip for Detecting Tuberculosis Drug Resistance Based on Polymerase Chain Reaction (PCR)-Magnetic Bead Molecule Platform

**DOI:** 10.3389/fmicb.2018.02106

**Published:** 2018-09-07

**Authors:** Jingtong Lyu, Wenjie Wu, Peng Cheng, Xun Liu, Fei Luo, Zehua Zhang, Kanglai Tang, Jianzhong Xu

**Affiliations:** Department of Orthopaedics, Southwest Hospital, Third Military Medical University, Chongqing, China

**Keywords:** tuberculosis, gene chip, drug resistance, sensibility, specificity

## Abstract

**Objective:** A Tag Array chip was used to detect plasmids of different template concentration, and then analyzed for sensitivity and specificity. Drug resistance genes from tuberculosis clinical specimens were detected, giving comparative phenotypic resistance results to explore the feasibility and value of clinical applications.

**Methods:** Twenty-four strains of *Mycobacterium Tuberculosis* (MTB) having sequence differences in extracted plasmids of mutant strains. The plasmid was diluted into different concentrations, and then was performed to analyze the sensitivity and specificity of the chip system. A total of 427 clinical specimens (including spinal tuberculosis and pulmonary tuberculosis) were collected from patients who came from seven hospitals. Design, optimization and preparation of the chip detection system, sequencing and phenotypic drug susceptibility results were analyzed to evaluate the sensitivity and specificity of the gene chip.

**Results:** In the template, concentrations of 1 × 10^3^ copies/μL and above were consistent with sequencing results in the mutant. The sensitivity and specificity in spine Tuberculosis specimen of rifampicin (RFP) were 94.40 and 92.86%; isoniazide (INH) were 92.37 and 87.50%; ethambutol (EMB) were 61.36 and 89.29%; fluoroquinolones (FQS) were 79.41 and 92.86%; streptomycin (SM) were 90.18 and 89.29%; second line drugs (SLD) were 77.61 and 83.93%. In Pulmonary Tuberculosis specimen, the sensitivity and specificity respectively were RFP: 92.74%; 93.75%; INH: 91.26%; 87.50%; EMB: 54.17%; 89.58%; FQS: 84.87%; 93.75%; SM: 86.73%; 85.42%; SLD: 80.9%; 91.67%. The RFP, INH, FQs and SM resistance genes was highly sensitive and specific: however, for detection of amikacin (AMK), capreomycin (CPM), kanamycin (KM), specificity was higher, but sensitivity was lower. Sensitivity for the detection of a mutation in the eis promoter region could be improved.

**Conclusion:** Tag Array chip can achieve rapid, accurate detection of tuberculosis resistance, which has important clinical significance and feasibility.

## Background

Progress in TB control is further hindered by the continued use of timeworn diagnostic methods. Although conventional drug sensitive test (DST) can provide definitive results and remains the gold standard method for tuberculosis detection, this testing method is extraordinarily time consuming and lead to many patients to go undiagnosed and untreated, allowing for continued spread of TB in the area. The delayed diagnosis may be bring about an incorrect or incomplete treatment. Defect of treatment is thought to be a driving forces behind the emergence of multidrug-resistant TB (MDR-TB) or extensively drug-resistant TB (XDR-TB), Moreover, DST is not conventionally used for spinal tuberculosis in most resource-poor hospitals in China because of biosafety concerns and inadequate infrastructure, which reveals key constraints for therapy of the disease. Therefore, the development of a rapid and accurate molecular detection of MDR-TB in clinical hospitals has become the focus of attention.

Recently, Nucleic acid amplification tests (NAAT) have become more common in the diagnosis of tuberculosis. Several commercial molecular test kits have been developed for the detection of gene mutations associated with MDR-TB (Boehme et al., 2011; Cabibbe et al., 2011; Denkinger and Pai, 2014; Javed et al., 2014; Li et al., 2015; Nathavitharana et al., 2016). However, Resistance of many SLD is still not rapidly detectable. Moreover, most of these detection products specifically amplify the genetic material of the causative organisms to detectable levels through PCR technology, and are unable to detect a large number of gene mutations.

As an integrated system, gene chip, fixed with thousands of functionalized probes, can achieve accurate, and rapid analysis for MDR-TB samples. Based on the detection of Amplification Refractory Mutation System-Polymerase Chain Reaction (ARMS-PCR)-magnetic bead Tag Array platform, we developed the Tag Array chip, allowing sensitive and specific detection. Moreover, specimens of spinal and pulmonary tuberculosis were analyzed to verify the effect in a clinical application.

## Materials and Methods

### Study Design and Specimens

Specific primer sequences are shown (**Supplementary Material 1**).

In order to obtain sufficient clinical samples, we organized six other hospitals (including three TB specialist hospitals) to work together for clinical sample collection. The achieved specimens after decontamination were preserved in a −30°C freezer for later analysis by the Tag Array chip. In order to avoid bias, specimens were excluded if the patient already received anti-TB drug treatment for more than 2 weeks before the sample collection. Finally, there were 421 specimens (234 sputum samples and 187 spinal samples). The Tuberculosis Drug Resistance Mutation Database (TBDReaMDB) was used to access to RFP, INH, EMB, fluoroquinolone ketone, aminoglycoside and cyclic peptide (amikacin, KM and CPM and other common drug resistance genes, mutations and mutation coverage (**Table 1**), covering a relatively wide selection of mutations.

**Table 1:** Resistance gene loci and coverage.

Drug	Drug resistance gene	Coverage rate(%)	Mutation site	Coverage rate(%)
Rifampin	rpoB	84%	511	1%
			513	
			516	3%
			522	1%
			526	20%
			531	55%
			533	4%
Isoniazid	katG	68%	315	51%
	inhA		-15	18%
	oa		−10	
			−39	
Streptomycin	rpsL	85%	43	72%
			88	5%
	rrs		513	7%
			516	1%
Ethambutol	embB	57%	306	57%
Fluoroquinolones	gyrA	92%	90	29%
			94	57%
			91	6%
Aminoglycosides/ Cyclic peptides (Amikacin, kanamycin and Aspergillus)	rrs	—	1401	
			1402	
			1484	

### Processing of Specimens and Mycobacteriology Analyses

All clinical specimens were decontaminated using the N-acetyl-L-cysteine (NALC) −NaOH method (18) with a final NaOH concentration of 1%. After concentration by centrifugation (20 min at 3,000 × *g*), the sediment was re-suspended in 1.5 ml of 0.5 M phosphate buffer (pH 6.8) and inoculated for culture on both Lowenstein-Jensen (LJ) solid media and the BACTEC MGIT 960 (Becton-Dickinson, Sparks, MD, United States) system, and the sensitivity test for eight kinds of anti-TB drugs proceeded with a modified absolute concentration method, according to the rules for the bacteriological diagnosis of tuberculosis (World Health Organization [WHO], 2011).

### DNA Sequencing of Drug Resistance-Associated Genes

Genomic DNA was isolated from bacteria cultured on L-J medium. A bacteria loop was suspended in water (500 μl) and heated at 95°C for 15 min. The DNA used for amplification by PCR was obtained by heat shock extraction (1 min at 95°C and 1 min on ice, repeated five times). A volume of 5 μl was used in PCR with the oligonucleotide primers described below. To detect RFP resistance, the rpoB gene were amplified and sequenced using primers 5′-AGGCGATCACACCGCAGACGT-3′ and 5′- GCCGATCAGACCGATGTTGG −3′. For detection of SM resistance, the rpsL gene were amplified and sequenced using primers 5′-CTGGTCCGCAAGGGTCGTC-3′ and 5′-CCCTGCGTCCAGCGAACC-3′. To detected INH resistance, the katG and inhA were amplified and sequenced using, respectively, primers 5′-CTCTTCGTCAGCTCCCACTCG-3′, and 5′- GTCGGCGGTCACACTTTCG-3′ and primers 5′-GGGTTTGGCCCCTTCAGTG-3′, and 5′-GCCTCGCTGCCCAGAAAG-3′. To detect EMB resistance, the embB gene were amplified and sequenced using primers 5′-CGTGGTGATATTCGGCTTCCTG-3′ and 5′-TGCCGAACCAGCGGAAATAG-3′. For detection of FQs resistance, the QRDRs of gyrA were amplified and sequenced using primers 5′-AGCATCTCCATCGCCAACGG-3′ and 5′-ACCGCAGCCACGCCAAGTC-3′. To detected the second-line anti-TB drugs (AMK, CPM, and KM) resistance, the rrs gene (positions 1401 to 1484) were amplified and sequenced using, respectively, primers 5′-AGAACCCCTCACGGCCTACG-3′ and 5′-GCAACGCTGCGGTGAATACG-3′. After amplification, unincorporated nucleotides and residual primers were filtered and removed (**Supplementary Material 2**).

### Tag Array Chip Assays

**FIGURE 1:**
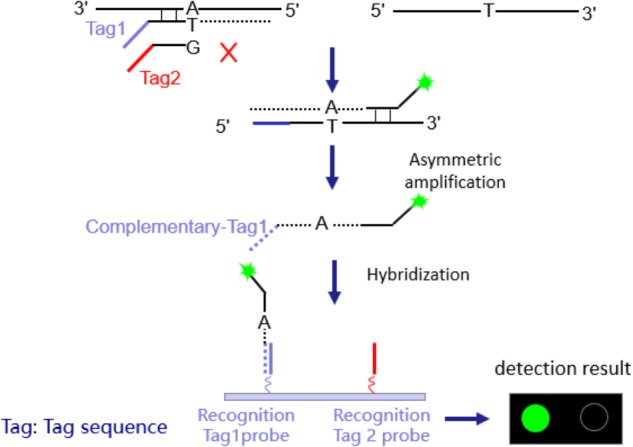
Schematic diagram of experimental principle.

The chip design see **Tables 2**, **3** and **Figure 1**. A visible colony was chosen, placed on the tube containing nucleic acid extraction buffer (100 μl: 5 mg/ml lysozyme, 2mg/ml Proteinase K, 1% Sodium dodecyl sulfate (SDS), potassium acetate, phenol/chloroform/isoamylol 25:24:1), oscillated for 5 min, then placed in a 95°C water bath for 5 min, and stored at −20°C. PCR fluorescence probe quantitatively analyzed the extracted nucleic acid. According to the concentration, samples were diluted or concentrated: the concentration of DNA were 1 × 10^2^ copies/μL (group 1), 1 × 10^3^ copies/μL (group 2), 1 × 10^4^ copies/μL (group 3), 1 × 105 copies/μL RFP (group 4), 5 × 105 copies/μL (group 5), 1 × 106 copies/μL (group 6). Each sample proceeded to eight tubes for PCR amplification. 25 μl amplification PCR reagents and 5 μl template DNA were added to each tube, in a total reaction volume of 30 μl for PCR amplification.

**Table 2:** Microarray probe design.

Probe species	Probe name	Probe meaning	Probe name	Probe meaning
Quality control probe	QC	SuFace chemical quality control probe	BC	Blank control probe
	PC	Hybrid positive control probe	MC	The Magnetic bead quality control probe
	gbTAG282	Quality control probe for *Mycobacterium tuberculosis*		
Gene site detection probe	gbTAG197	gyrA90 Mutant probe	gbTAG235	oa-10 Mutant probe
	gbTAG264	gyrA91 Mutant probe	gbTAG217	rrs1401 Mutant probe
	gbTAG200	gyrA94 Mutant probe	gbTAG279	rrs1402 Mutant probe
	gbTAG275	embB306 Mutant probe	gbTAG205	rrs1484 Mutant probe
	gbTAG168	embB306 Mutant probe	gbTAG171	katG315 Mutant probe
	gbTAG198	katG315 Mutant probe	gbTAG245	rpoB511 Mutant probe
	gbTAG206	inhA-15 Mutant probe	gbTAG257	rpoB513 Mutant probe
	gbTAG104	rpsL43 Mutant probe	gbTAG209	rpoB516 Mutant probe
	gbTAG221	rpsL88 Mutant probe	gbTAG108	rpoB522 Mutant probe
	gbTAG270	rrs513 Mutant probe	gbTAG277	rpoB526 Mutant probe
	gbTAG301	rrs516 Mutant probe	gbTAG273	rpoB531 Mutant probe
	gbTAG202	oa-39 Mutant probe	gbTAG260	rpoB533 Mutant probe

**Table 3:** Microarray probe design description.

	1–3	4–6	7–9	10–12
1	QC_HEX	BC	MC	EC
2	gbTAG197	gbTAG270	gbTAG245	
3	gbTAG264	gbTAG301	gbTAG257	
4	gbTAG200	gbTAG202	gbTAG209	
5	gbTAG275	gbTAG235		
6	gbTAG168	gbTAG217	gbTAG108	
7	gbTAG198	gbTAG279	gbTAG277	
8	gbTAG206	gbTAG205	gbTAG273	gbTAG282
9	gbTAG104	gbTAG171	gbTAG260	
10	gbTAG221			
11	EC	MC	BC	QC_HEX

The eight PCR reactions of each sample were combined into two centrifuge tubes. The reaction conditions are shown in **Supplementary Materials 3**. 40 μl of the prepared magnetic bead suspensions were added, and stood. The mixture was placed onto the magnetic frame, and adsorbed for 15 s. After added 60 μl 0.1 N NaOH, the tube was adsorbed for 15 s on the magnetic frame. With 40 μl hybridization buffer, the centrifuge tube adsorbed for 15 s again, next added to the hybrid buffer for hybridization. Each purified sample was added to two microarrays, and the hybrid reaction mixture was slowly injected through the sample-adding hole, until the chip was covered (about 15.5 μl). Then the sealed chip was placed inside to initiate the hybridization reaction (50°C, 1 h), and read the signal using a microarray chip scanner (LuxScan 10K-B). The detection time is 6–8 h.

### Data Analysis

The chip test results were compared with previous sequencing results to evaluate the accuracy of gene detection. These results were also compared with bacterial culture and the results of the absolute concentration method for drug susceptibility testing. In cases of discrepant results, the medical history, clinical feature, and histopathological examination of patients were included in the final evaluation.

The specificity (The true negative rate: the probability of actual resistance was proved to be resistant by culture + DST), sensitivity (The true positive rate: The probability that the drug is actually non-resistant and is proved to be non-resistant by culture + DST), and the accuracy (the degree to which the observed value corresponds to the standard value) of the two generations of drug-resistant TB chips were evaluated.

This research was approved by the Ethics Committee of the First Affiliated Hospital of the Third Military Medical University, People’s Liberation Army.

## Results

### Phenotypic Drug Sensitivity of *Mycobacterium tuberculosis*

Spinal Tuberculosis: 67 aminoglycosides (AMK, CPM, KM) resistant strains, 44 EMB resistant strains, 112 SM resistant strains, 118 INH resistant strains, and 126 rifampicin resistant strains. Pulmonary Tuberculosis: 89 aminoglycosides (AMK, CPM, KM) resistant specimen, 72 ethambutol resistant specimen, 98 streptomycin resistant specimen, 103 isoniazid resistant specimen, and 124 rifampicin resistant specimen. The above gene loci were previously sequenced.

### Comparison of Chip Scanning and Phenotypic Drug Results, and Analysis Sensitivity and Specificity

1. Spinal Tuberculosis: Among 126 RFP resistant phenotype strains, 119 strains were identified to have rpoB gene mutations; in phenotypically sensitive strains, four were identified as having rpoB gene mutations. The mutation frequency of rpoB531 was 78 strains (60.93%), rpoB516 was 20 strains (15.63%), rpoB513 was nine strains (7.03%), rpoB511 was nine strains (7.03%), rpoB511 was six strains (4.69%), and 11 strains had multiple mutations in the rpoB gene. The sensitivity and specificity of rifampicin (RFP) were 94.40 and 92.86%. Of 118 strains that were INH resistant, 109 strains were detected as having phenotypic resistance; whereas, in sensitive strains, 13 strains were detected. The mutation frequency of KatG315 was 105 strains (86.07%), inhA −15 was 31 strains (25.41%), oxyR-ahpC was six strains (4.92%). There are 21 strains that shared KatG315 and inhA −15 mutations, but no oxyR-ahpC gene mutation was associated with other strains. The sensitivity and specificity of INH were 92.37 and 87.50%.

Among the 44 strains of EMB resistant phenotypes, there were 27 strain mutations in the embB306 site, and embB306 mutation was detected in six phenotypically sensitive strains. Mutations occurred in the gyrA gene in 81 strains of 102 strains of quinolone resistant strains; in sensitive strains, four strains had the gyrA gene mutation. Overall, the mutation rate from high to low for gyrA94 was 55 strains (64.71%), gyrA90 was 25 strains (29.42%), and gyrA91 was four strains (4.71%). In 112 strains of SM resistant strains phenotype, the chip detected 101 strains of resistant genes in mutation sites; at the same time, in sensitive strains, 12 strains exhibited mutations. The mutation frequency for rpsL 43 was 91 strains (80.53%), rpsL88 was 16 strains (14.16%), rrs513 was six strains (5.31%), and rrs516 was two strains (1.77%). The sensitivity and specificity of EMB. FQs and SM were 61.36%, 89.29%; 79.41%, 92.86%; 90.18%, 89.29%

2. Pulmonary Tuberculosis: Among 124 rifampin resistant phenotype strains, 115 strains were identified to have rpoB gene mutations; in phenotypically sensitive strains, three were identified as having rpoB gene mutations. The mutation frequency of rpoB531 was 66.94%, rpoB516 was 18.18%, rpoB513 was 6.61%, rpoB511 was 4.13%, rpoB511 was 1.65%, rpoB511 was 2.48%, and 14 strains had multiple mutations in the rpoB gene. Of 103 isoniazid phenotypic resistant strains, 94 strains were detected as having gene mutation; in phenotypic sensitive strains, six strains were detected. The mutation frequency of KatG315 was 80.20%, inhA −15 was 12.87%, oxyR-ahpC was 6.93%. There are 24 strains that shared KatG315 and inhA −15 mutations, but no oxyR-ahpC gene mutation was associated with other strains.

Among the 72 strains of ethambutol resistant phenotypes, there were 39 strain mutations in the embB306 site, and five embB306 mutation was detected in phenotypically sensitive strains. Mutations occurred in the gyrA gene in 101 strains of quinolone resistant strains; in sensitive strains, three strains had the gyrA gene mutation. Overall, the mutation rate from high to low for gyrA94 was 66.36%, gyrA90 was 26.17%, and gyrA91 was 7.48%. In streptomycin resistant strains phenotype, the chip detected 85 strains of resistant genes in mutation sites; in sensitive strains, seven strains exhibited mutations. The mutation frequency for rpsL 43 was 86.92%, rpsL88 was 9.35%, rrs513 was 1.87%, and rrs516 was 1.87%. The sensitivity and specificity respectively were RFP: 92.74%; 93.75%; INH: 91.26%; 87.50%; EMB: 54.17%; 89.58%; FQS: 84.87%; 93.75%; SM: 86.73%; 85.42%.

**FIGURE 2:**
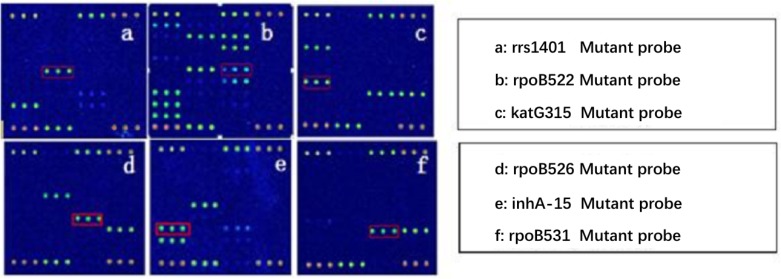
Test results of the experiment–Part 1.

**FIGURE 3:**
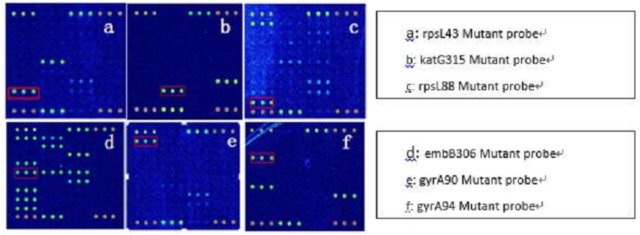
Test results of the experiment–Part 2.

Because AMK, CPM, KM possess cross resistance, the results were combined when analyzed (clinical isolates, as long as there was resistance to one or more drug, named resistant strains). Spinal Tuberculosis: In 67 phenotypically resistant strains, 52 strains had mutations; of the sensitive strains, 11 strains had rrs1401 gene mutations. The frequency of mutation for rrs1401 was 62 strains (98.41%), rrs1484 was one strain (1.59%), and rrs1402 was no strains (0.00%). Pulmonary Tuberculosis: In 89 phenotypically resistant strains, 72 strains had mutations; of the sensitive strains, four strains had rrs1401 gene mutations. The frequency of mutation for rrs1401 was 96.05%, rrs1484 was 3.95%, and rrs1402 was 0.00%. The sensitivity and specificity of SLD in spinal and pulmonary tuberculosis were 77.61%, 83.93%, 80.9%, 91.67%. Detailed results are illustrated in **Tables 4**, **5**. Hybridization results are shown in **Figures 2**, **3**.

**Table 4:** Sensitivity, specificity and consistency of chip detection and phenotypic resistance (Spinal tuberculosis strains).

Anti-tuberculosis drugs	Sensitivity (%)	Specificity (%)	Accuracy (%)
RMP	94.40[91.06–97.74]	92.86[89.12–96.60]	93.96[90.46–97.42]
INH	92.37[88.43–96.31]	87.50[82.6–92.41]	90.80[86.51–95.09]
EMB	61.36[51.82–70.90]	89.29[83.23–95.35]	77.00[68.75–85.25]
SM	90.18[85.68–94.68]	89.29[84.61–93.97]	89.88[85.32–94.44]
fQs	79.41[73.10–85.72]	92.86[88.84–96.88]	84.18[78.49–89.87]
SLID	77.61[75.28–79.94]	83.93[77.44–90.42]	80.49[73.49–87.49]

**Table 5:** Sensitivity, specificity and consistency of chip detection and phenotypic resistance (Pulmonary tuberculosis strains).

Anti-tuberculosis drugs	Sensitivity (%)	Specificity (%)	Accuracy (%)
RMP	92.74[88.86–96.62]	93.75[90.13–97.37]	93.02[89.21–96.83]
INH	91.26[86.76–95.76]	87.50[84.81–90.19]	90.07[88.34–93.06]
EMB	54.17[45.26–63.08]	89.58[84.11–95.05]	68.33[60.01–76.65]
SM	86.73[81.23–92.23]	85.42[79.70–91.14]	86.30[80.72–91.88]
fQs	84.87[79.44–90.30]	93.75[90.08–97.42]	87.43[82.40–92.46]
SLID	80.90[74.32–87.48]	91.67[87.04–96.30]	84.67[78.64–90.7]

*The sensitivity and specificity of gene chip was based on the improved absolute concentration of drug sensitivity method. []: 95% CI.*

## Discussion

Today, TB is still the main reason of leading death in the worldwide. Strengthening laboratory diagnostic is an important part of controlling TB epidemic. However, conventional DST for M. tuberculosis still relies on culture of the bacilli and requires a minimum of several weeks, therefore, ineffective anti-TB regimens may cause acquired drug resistance and local recurrence during this period (Rüsch et al., 2006). Moreover, DST for TB spondylitis is not performed routinely in most resource-poor hospitals in China because of the biosafety concerns and inadequate infrastructure, which present a major hindrance for the treatment of the disease. Therefore, rapid, accurate detection and early diagnosis is necessary for the successful management of MDR-TB in China (Gu et al., 2015).

In addition to genotype, MTBDRsl can simultaneously detect resistance to FQS, amikacin, kanamycin (KM), capreomycin (CPM), EMB. Other products detect only RIF and or INH resistance. Resistance in many second-line drugs is still not rapidly detectable. A Tag Array gene chip takes around four to 6 h to detect TB drug resistance. It is important for doctors to rapidly diagnose tuberculosis early and process individualized chemotherapy.

Tag Array TB chip, can synchronously detect the total 38 mutants of 22 mutant sites, and can be used for detecting resistance to RFP, INH, EMB, SM, AMK/CPM/KM, FQS, etc. Furthermore, in an emergency or extreme cases, the visual chip can be estimated result by eyes, which reduces experimental conditions.

Some studies have reported the rpoB gene mutation rate to be 96-100% in RFP resistant *Mycobacterium tuberculosis* (Hunt et al., 1994; Tan et al., 2012). A high frequency of mutation sites occurred in rpoB531 and rpoB526, which was consistent with experimental results (Hazbón et al., 2006). This study selected the rpoB gene to represent RFP resistance detection, and the sensitivity was 94.40% (spine) and 92.74% (pulmonary), whereas the specificity was 92.86% (spine) and 93.75% (pulmonary). The result of the detection analysis was in the same levels of what has been reported from other commercial NAATs (Hunt et al., 1994; Hazbón et al., 2006; Tan et al., 2012).

Mutations in the KatG gene confer high level INH resistance. S315T genetic mutations mainly caused decreased INH substrate affinity at the expense of only modest reduction in overall catalase activity. At the same time, the mutations in the inhA (associated with low-level INH resistance and cross-resistance to ethionamide) and ahpC genes are closely related to INH resistance (Wilson and Collins, 1996; Huang et al., 2009). In the United States, the sensitivity of detecting mutations in INH resistance is close to 100% (Campbell et al., 2011; Lin and Desmond, 2014; Lin et al., 2014), but some study observed that the rate of INH resistance are related to location, so affecting the sensitivity of related gene mutation detection methods (Abal et al., 2002; Mokrousov et al., 2002; Zhao et al., 2014). Linger et al. detected MDR-TB in pulmonary tuberculosis using simplified microarray system, they observed that the sensitivity for INH was 90.0%, and the specificity was 98.2% (Linger et al., 2014). Gu et al. (2015) reported the detection efficiency of GenoType MTBDRplus in bone and joint tuberculosis, the sensitivity was 85.71%, and the specificity was 100% for INH resistance. In our research, the chip detection sensitivity was 92.37% (spine) and 91.26% (pulmonary), the specificity was 87.50% (spine) and 87.50% (pulmonary).

Ethambutol is combined with INH, rifampicin and pyrazinamide in standard chemotherapy, and the resistance rate is lower than SM (Campbell et al., 2011). Previous studies have shown that the embB gene, especially the codon embB306 mutation, is the main mechanism of EMB resistance, and also includes the 319, 354, 406 mutation sites (Telenti et al., 1997). Recently, there have been reports of false positive and false negative results in the molecular diagnosis of tuberculosis drug resistance. This was related to the coverage of resistant sites, undiscovered resistance sites and mechanisms of drug resistance (Ramaswamy et al., 2000; Lee et al., 2004). However, some reports showed that only 28-68% of EMB resistant tuberculosis contains the embB306 mutation (Plinke et al., 2006; Huang et al., 2009), and the sensitivity to molecular diagnostic testing of EMB resistance was 60-80%, and the specificity was 93-100% (Sirgel et al., 2012). Our chip detection results were consistent with previously reported data, showing high specificity, but poor sensitivity. According to the data analysis, we believe that the possibility of phenotypic EMB resistance is very large when the chip gives a positive result; but that negative chip results have little reference value and may also occur with phenotypic resistance.

Levofloxacin, moxifloxacin, and gatifloxacin are commonly used anti-tuberculosis second-line drugs. They target the DNA gyrase, and is coded for by 2 genes, gyr A and B. When mutations occur within the gyrA region, which determines resistance (most commonly gyrA94 and gyrA90 mutations), it will result in different levels of quinolone resistance (Huang et al., 2011). Some studies, using linear probe analysis, pyrosequencing, or Sanger sequencing detected quinolone resistance and compared it with phenotypic resistance. The results showed that sensitivity was 80% and specificity was 100% (Ramaswamy et al., 2000; Lin et al., 2014). In our research, the sensitivity was 79.41% (spine) and 84.87% (pulmonary), the specificity was 92.86% (spine) and 93.75% (pulmonary). The results were consistent with previously reported data, showing high specificity, but poor sensitivity.

In China, SM resistance mutations are very high, mainly because SM has been applied as the first-line anti tuberculosis drug there for many years. The resistance variable sites are located in rpsL43, rpsL88, rrs531, rrs516. The TBDReaMDB database shows that these four mutation sites cover 85% SM resistant tuberculosis, including rpsL43 (72%), rpsL88 (5%), rrs531 (7%), rrs516 (1%) (Tracevska et al., 2004). The sensitivity of our chip was 90.18% (spine) and 86.73% (pulmonary), specificity was 89.29% (spine) and 85.42% (pulmonary), yet this still has a high detection efficiency.

Amikacin and CPM cross resistance is caused by mutations in the rrs1400 region, and mainly appear in the 1401, 1402, 1484 sites (Jugheli et al., 2009; Georghiou et al., 2012; Tagliani et al., 2015). RrsA1401G mutations can lead to KM (60%), AMK and Aspergillus (75%) resistance; therefore, this site mutation should be used as a drug resistance marker of the three drugs (Du et al., 2013). It has been reported that the rrsA1401G mutation was found to be resistant to KM (100%), AMK (100%) and CPM (63%). In addition, rrs1402 and 1484 mutations also occurred in a similar cross resistance situation (Jnawali et al., 2013). Furthermore, the sequence was unable to achieve an accurate assessment of the resistance of KAN, AMK and CAP respectively. A high rate of incidence of cross resistance in the rrs1400 region was also undeterminable. if a common site mutation is discovered, the three drugs should be excluded from chemotherapy. In this research, we judged drug resistance as phenotypic resistance including one antibiotic, which improved the effectiveness of early chemotherapy and avoided ineffective chemotherapy. The literature reported that for molecular detection of the rrs1400 mutation, compared with phenotypic detection, the sensitivity was 60.4%, and specificity was 100%. For GenoType MTBDRsl, the sensitivity was 86.4%, and the specificity was 90.1% (Du et al., 2013). Our research found that chip sensitivity was 77.61% (spine) and 80.9% (pulmonary), and specificity was 83.93% (spine) and 91.67% (pulmonary). The sensitivity was lower than the GenoType MTBDRsl assay, which may be related to no detection in the eis promoter region.

## Conclusion

The detection condition of this chip is not high, allowing to be used in any lab that uses the traditional method, hence, it is easy to popularize in the developing countries. Compared with other currently available detected methods, this method is less cost and time-consuming, more detected drugs and genetic site than both Bactec 960 TB and other molecule detected methods, and correlates very well as predictor of resistance. The EMB resistance gene has low sensitivity, and it is therefore easy to misdiagnose; however, its specificity is high, which provides an important reference value. We believe this gene chip is promising for future testing for reduces difficulties of the traditional detected methods, while improving accurate testing of MDR-TB or XDR-TB.

## Author Contributions

JL, WW, KT, and JX carried out the molecular genetic studies, participated in the sequence alignment, and drafted the manuscript. ZZ and FL participated in its design and coordination and helped to draft the manuscript. XL performed the statistical analysis, drafted the manuscript, and helped to polish the language. PC carried out the microarrays detection and participated in the sequence alignment and participated in the design of the study. JX conceived of the study, participated in its design, and provided theoretical and technical support. All authors read and approved the final manuscript.

## Conflict of Interest Statement

The authors declare that the research was conducted in the absence of any commercial or financial relationships that could be construed as a potential conflict of interest.

## References

[B1] Abal A. T. Ahmad S. Mokaddas E. (2002). Variations in the occurrence of the S315T mutation within the katG gene in isoniazid-resistant clinical Mycobacterium tuberculosis isolates from Kuwait. *Microb. Drug Resist.* 8 99–105. 10.1089/107662902760190644 12118524

[B2] Boehme C. C. Nicol M. P. Nabeta P. (2011). Feasibility, diagnostic accuracy, and effectiveness of decentralised use of the Xpert MTB/RIF test for diagnosis of tuberculosis and multidrug resistance: a multicenter implementation study. *Lancet* 377 1495–1505. 10.1016/S0140-6736(11)60438-8 21507477 PMC3085933

[B3] Cabibbe A. M. Miotto P. Lazzeri E. Mugasa J. Santoro F. (2011). New DNA microarray platform for detection of MDR Mycobacterium tuberculosis and of drug-resistant malaria. *Clin. Microbiol. Infect.* 17(Suppl. 3),591–592.

[B4] Campbell P. J. Morlock G. P. Sikes R. D. Dalton T. L. Metchock B. Starks A. M. (2011). Molecular detection of mutations associated with first- and second-line drug resistance compared with conventional drug susceptibility testing of Mycobacterium tuberculosis. *Antimicrob. Agents Chemother.* 55 2032–2041. 10.1128/AAC.01550-10 21300839 PMC3088277

[B5] Denkinger C. M. Pai M. (2014). Using cerebrospinal fluid for the diagnosis of tuberculous meningitis with genexpert. *Eur. Respir. J.* 44 1095–1096. 10.1183/09031936.00106914 25271230

[B6] Du Q. Dai G. Long Q. Yu X. Dong L. Huang H. (2013). Mycobacterium tuberculosis rrs A1401G mutation correlates with high-level resistance to kanamycin, amikacin, and capreomycin in clinical isolates from mainland China. *Diagn. Microbiol. Infect. Dis.* 77 138–142. 10.1016/j.diagmicrobio.2013.06.031 23948547

[B7] Georghiou S. B. Magana M. Garfein R. S. Catanzaro D. G. Catanzaro A. Rodwell T. C. (2012). Evaluation of genetic mutations associated with Mycobacterium tuberculosis resistance to amikacin, kanamycin and capreomycin: a systematic review. *PLoS One* 7:e33275 10.1371/journal.pone.0033275 PMC3315572 22479378

[B8] Gu Y. Wang G. Dong W. Li Y. Ma Y. Shang Y. (2015). Xpert MTB/RIF and GenoType MTBDRplus assays for the rapid diagnosis of bone and joint tuberculosis. *Int. J. Infect. Dis.* 36 27–30. 10.1016/j.ijid.2015.05.014 26004172

[B9] Hazbón M. H. Brimacombe M. Bobadilla del Valle M Cavatore M. Guerrero M. I. Varma-Basil M. (2006). Population genetics study of isoniazid resistance mutations and evolution of multidrug-resistant Mycobacterium tuberculosis. *Antimicrob. Agents Chemother.* 50 2640–2649. 10.1128/AAC.00112-06 16870753 PMC1538650

[B10] Huang W.-L. Chen H.-Y. Kuo Y.-M. Jou R. (2009). Performance assessment of the Geno-Type MTBDRplus test and DNA sequencing in detection of multidrug-resistant Mycobacterium tuberculosis. *J. Clin. Microbiol.* 47 2520–2524. 10.1128/JCM.02499-08 19494067 PMC2725636

[B11] Huang W. L. Chi T. L. Wu M. H. Jou R. (2011). Performance assessment of the GenoType MTBDRsl test and DNA sequencing for detection of second-line and ethambutol drug resistance among patients infected with multidrug-resistant Mycobacterium tuberculosis. *J. Clin. Microbiol.* 49 2502–2508. 10.1128/JCM.00197-11 21562102 PMC3147822

[B12] Hunt J. M. Roberts G. D. Stockman L. Felmlee T. A. Persing D. H. (1994). Detection of a genetic locus encoding resistance to RFP in mycobacterial cultures and in clinical specimens. *Diagn. Microbiol. Infect. Dis.* 18 219–227. 10.1016/0732-8893(94)90024-8 7924218

[B13] Javed N. Aslam M. Mushtaq M. A. Khan T. Shaheen M. Z. (2014). Role of gene Xpert in diagnosis of tuberculous pleural effusion: comparison with pleural biopsy. *Eur. Respir. J.* 44 2655.

[B14] Jnawali H. N. Hwang S. C. Park Y. K. Kim H. Lee Y. S. Chung G. T. (2013). Characterization of mutations in multi- and extensive drug resistance among strains of Mycobacterium tuberculosis clinical isolates in Republic of Korea. *Diagn. Microbiol. Infect. Dis.* 76 187–196. 10.1016/j.diagmicrobio.2013.02.035 23561273

[B15] Jugheli L. Bzekalava N. de Rijk P. Fissette K. Portaels F. Rigouts L. (2009). High level of cross-resistance between kanamycin, amikacin, and CPM among Mycobacterium tuberculosis isolates from Georgia and a close relation with mutations in the rrs gene. *Antimicrob. Agents Chemother.* 53 5064–5068. 10.1128/AAC.00851-09 19752274 PMC2786337

[B16] Lee A. S. G. Othman S. N. K. Ho Y. M. Wong S. Y. (2004). Novel mutations within the embB gene in ethambutol-susceptible clinical isolates of Mycobacterium tuberculosis. *Antimicrob. Agents Chemother.* 48 4447–4449. 10.1128/AAC.48.11.4447-4449.2004 15504879 PMC525425

[B17] Li R. Ruan Y. Sun Q. Wang X. Chen M. Zhang H. (2015). Effect of a comprehensive programme to provide universal access to care for sputum-smear-positive multidrug-resistant tuberculosis in China: a before-and-after study. *Lancet Glob. Health* 3 e217–e228. 10.1016/S2214-109X(15)70021-5 25794675

[B18] Lin S. Y. G. Desmond E. P. (2014). Molecular diagnosis of tuberculosis and drug resistance. *Clin. Lab. Med.* 34 297–314. 10.1016/j.cll.2014.02.005 24856529

[B19] Linger Y. Kukhtin A. Golova J. Perov A. Lambarqui A. Bryant L. (2014). Simplified microarray system for simultaneously detecting rifampin, isoniazid, ethambutol, and streptomycin resistance markers in Mycobacterium tuberculosis. *J. Clin. Microbiol.* 52 2100–2107. 10.1128/JCM.00238-14 24719444 PMC4042730

[B20] Lin S. Y. Rodwell T. C. Victor T. C. Rider E. C. Pham L. Catanzaro A. (2014). Pyrosequencing for rapid detection of extensively drug-resistant Mycobacterium tuberculosis in clinical isolates and clinical specimens. *J. Clin. Microbiol.* 52 475–482. 10.1128/JCM.01821-13 24478476 PMC3911348

[B21] Mokrousov I. Otten T. Filipenko M. Vyazovaya A. Chrapov E. Limeschenko E. (2002). Detection of isoniazid-resistant Mycobacterium tuberculosis strains by a multiplex allele-specific PCR assay targeting katG Codon 315 variation. *J. Clin. Microbiol.* 40 2509–2512. 10.1128/JCM.40.7.2509-2512.2002 12089271 PMC120554

[B22] Nathavitharana R. R. Hillemann D. Schumacher S. G. Schlueter B. Ismail N. Omar S. V. (2016). Multicenter noninferiority evaluation of Hain GenoType MTBDRplus version 2 and Nipro NTM+MDRTB line probe assays for detection of RFP and isoniazid resistance. *J. Clin. Microbiol.* 54 1624–1630. 10.1128/JCM.00251-16 27076658 PMC4879293

[B23] Plinke C. Rusch-Gerdes S. Niemann S. (2006). Significance of mutations in embB codon 306 for prediction of ethambutol resistance in clinical Mycobacterium tuberculosis isolates. *Antimicrob. Agents Chemother.* 50 1900–1902. 10.1128/AAC.50.5.1900-1902.2006 16641474 PMC1472212

[B24] Ramaswamy S. V. Amin A. G. Gsel S. Stager C. E. Dou S. J. El Sahly H. (2000). Molecular genetic analysis of nucleotide polymorphisms associated with ethambutol resistance in human isolates of Mycobacterium tuberculosis. *Antimicrob. Agents Chemother.* 44 326–336. 10.1128/AAC.44.2.326-336.2000 10639358 PMC89679

[B25] Rüsch S. Gaby E. Casal M. (2006). Multicenter laboratory validation of the BACTEC MGIT 960 technique for testing susceptibilities of mycobacterium tuberculosis to classical second-line drugs and newer antimicrobials. *J. Clin. Microbiol.* 44 688–692. 10.1128/JCM.44.3.688-692.2006 16517840 PMC1393114

[B26] Sirgel F. A. Warren R. M. Streicher E. M. Victor T. C. van Helden PD Böttger E. C. (2012). gyrA mutations and phenotypic susceptibility levels to ofloxacin and moxifloxacin in clinical isolates of Mycobacterium tuberculosis. *J. Antimicrob. Chemother.* 67 1088–1093. 10.1093/jac/dks033 22357804

[B27] Tagliani E. Cabibbe A. M. Miotto P. Borroni E. Toro J. C. Mansjö M. (2015). Diagnostic performance of the new version (v2.0) of GenoType MTBDRsl assay for detection of resistance to fluoroquinolones and second-line injectable drugs: a multicenter study. *J. Clin. Microbiol.* 53 2961–2969. 10.1128/JCM.01257-15 26179309 PMC4540937

[B28] Tan Y. Hu Z. Zhao Y. Cai X. Luo C. Zou C. (2012). The beginning of the rpoB gene in addition to the RFP resistance determination region might be needed for identifying RFP/rifabutin cross-resistance in multidrug-resistant Mycobacterium tuberculosis isolates from Southern China. *J. Clin. Microbiol.* 50 81–85. 10.1128/JCM.05092-11 22075601 PMC3256682

[B29] Telenti A. Philipp W. J. Sreevatsan S. Bernasconi C. Stockbauer K. E. Wieles B. (1997). The emb operon, a unique gene cluster of Mycobacterium tuberculosis involved in resistance to ethambutol. *Nat. Med.* 3 567–570. 10.1038/nm0597-567 9142129

[B30] Tracevska T. Jansone I. Nodieva A. Marga O. Skenders G. Baumanis V. (2004). Characterisation of rpsL, rrs and embB mutations associated with streptomycin and ethambutol resistance in Mycobacterium tuberculosis. *Res. Microbiol.* 155 830–834. 10.1016/j.resmic.2004.06.007 15567277

[B31] Wilson T. M. Collins D. M. (1996). ahpC, a gene involved in isoniazid resistance of the Mycobacterium tuberculosis complex. *Mol. Microbiol.* 19 1025–1034. 10.1046/j.1365-2958.1996.449980.x 8830260

[B32] World Health Organization [WHO] (2011). *Global Tuberculosis Control: WHO Report*. Geneva: World Health Organization.

[B33] Zhao L. L. Sun Q. Zeng C. Y. Chen Y. Zhao B. Liu H. C. (2014). Molecular characterisation of extensively drug-resistant Mycobacterium tuberculosis isolates in China. *Int. J. Antimicrob. Agents* 45 137–143. 10.1016/j.ijantimicag.2014.09.018 25465521

