# Supplementary material for: A Chip for Detecting Tuberculosis Drug Resistance Based on Polymerase Chain Reaction (PCR)-Magnetic Bead Molecule Platform
Source: Front Microbiol. 2018 Sep 7;9:2106. doi: 10.3389/fmicb.2018.02106 (PMC6143819; doi:10.3389/fmicb.2018.02106)
Supplement: Supplementary file 1 [file Table_1.docx]

**Primer sequences of each system**

| system 1 | rpoB-F57-BT | CGGGAGCGGATGACCACCCAGGAC |
| --- | --- | --- |
|  | T257rB13CCR-2c | actccccgacacgagatatgagGTTGTTCTGGTCCATGAAcG |
|  | T209rB16TAR-5a | accttacgttcgacactcctctGACAGCGGGTTGTTCaGGTA |
|  | T260rB33CCR-4t | gtttccctactattcgcgtgttACAGACtGCtGGGCtCCG |
|  | rpsLF02-BT | CATGGCCGACAAACAGA |
|  | T104rL43AGR-5a | gtcgcgtaagattcggtcagtcAGCGCCGAGTTCGGaTTCC |
|  | T221rL88AGR-2t | gtgagggcggtcgatacactatGCACACCAGGCAGGTCtC |
|  | TBR-HGA2201_S-BT | GCCGGGTGCTCTATGCAAT |
|  | T197gA90GTR-3t | gtctccgcacgcaatgtttagtCAGGGTGTCGTAGATCGtCA |
|  | T264gA91CCR-2t | attccgctgtagggttcgtatgACCAGGGTGTCGTAGATCtG |
| system 2 | rpoB-F57-BT | CGGGAGCGGATGACCACCCAGGAC |
|  | T245rB11CCR-18-4a | ctgctcgttattagtgcttcgcGGTCCATGAATTGGaTCG |
|  | T273rB31TGRz-5a | tgccgcgaaatcacactttacaCCGtCGGGtCCCAGaGCCC |
|  | rrs513F22BT | GCCTGGGAAACTGGGTCTA |
|  | T301r516R-3t | aggtatcgccgcaaatctatccGCACCCTACGTATTACCtCA |
|  | T270r513(A-C)-21R-4a | actttcgcccgaactacaacagACCCTACGTATTACCGCaGCG |
| system 3 | rpoB-F57-BT | CGGGAGCGGATGACCACCCAGGAC |
|  | T257rB13AAR-2c | actccccgacacgagatatgagGTTGTTCTGGTCCATGAATcT |
|  | T209rB16GTR-3a | accttacgttcgacactcctctCGACAGCGGGTTGTTCTaGA |
|  | T209rB16GGR-2a | accttacgttcgacactcctctGACAGCGGGTTGTTCTGaC |
|  | r1401F08-BT | CGCGAGGTTAAGCGAATC |
|  | T279r1402(C-T)R-21-2g | gacatttagttccgctcgcttaGTGTTACCGACTTTCATGAgA |
|  | T205r1484(G-T)R | acctatctgcggactactacgagACGGCTACCTTGTTACGACTTA |
|  | inhAF05-BT /inhAF12-BT | GATCCGTCATGGTCGAAGTGTG |
|  | T206iA-15(C-T)R-3c | gactcgacaggacttcaaatggAGTCAtCCCGACAACCTAcCA |
| system 4 | rpoB-F57-BT | CGGGAGCGGATGACCACCCAGGAC |
|  | T108rB22TTR-2a | cagtcgccgtatagagcatagaaGCTTGTGGGTCAACCCaA |
|  | T277rB26CGR-2a | gtccaacggtcacgctaattttCCGACAGTCGGCGCTTaC |
|  | embBR16-1-BT | AGCGCCAGCAGGTTGTAATAC |
|  | T275eB306CTL-2a | acgtgacataatcgaaccctgcGACGGCTACATCCTGaGCG |
|  | T168eB306TCL-3t | gacggacgcatcggaaatcaaaCGGCTACATCCTGGGCtTC |
|  | r1401F08-BT | CGCGAGGTTAAGCGAATC |
|  | T217r1401(A-G )R-2t | ctccgacagacaatcgctaccaGTGTTACCGACTTTCATGACtC |
| system 5 | rpoB-R17-1-BT | CGAGCCGATCAGACCGAT |
|  | T277rB26GAL4t | gtccaacggtcacgctaattttGCTGTCGGGGTTGtCCG |
|  | T277rB26TAL-3g | gtccaacggtcacgctaattttGCTGTCGGGGTTGAgCT |
|  | oaF03BT | GACTGGCTCATATCGAGAATGC |
|  | T202oa-39R3t | tctgctcaagtccgggttagtcAGTCGCTGTCAGGCAAAGtTA |
|  | T235oa-10(C-T)R-3a | acgacactcacacggggtattaTTGGTTGCGACATTCCAaCA |
| system 6 | gyrAWT-R6-BT | CGACCAGGGCTGGGCCATGC |
|  | T200gA94GGL-2t | aagtgccgggtatctaacgtatGCGACGCGTCGATCTACtG |
|  | T200gA94GCL-3t | aagtgccgggtatctaacgtatGCGACGCGTCGATCTAtGC |
|  | T200gA94TAL-3c | aagtgccgggtatctaacgtatGCGACGCGTCGATCTcCT |
|  | katGR26+1-BT | CCGTCCTTGGCGGTGTATT |
|  | T198kG315ACL-3g | aacgtgcggataagactcggaaGGTAAGGACGCGATCACgAC |
|  | T171kG315AAL-3a | tatccctactcgttgcatgacaGGTAAGGACGCGATCACaAA |
| system 7 | rpoB-R17-1-BT | CGAGCCGATCAGACCGAT |
|  | T277rB26CTL-3t | gtccaacggtcacgctaattttGCTGTCGGGGTTGACtCT |
|  | T273rB31TTL-3c | tgccgcgaaatcacactttacaACCCACAAGCGCCGACTcTT |
|  | embBR16-1-BT | AGCGCCAGCAGGTTGTAATAC |
|  | T168eB306TAL-2a | gacggacgcatcggaaatcaaaACGGCTACATCCTGGGCAaA |
|  | is6110-4R-BT | AGGCGTCGGTGACAAAGG |
|  | T282is6110L-2 | caacgcttacgtccttacatcgTACGGTGCCCGCAAAGTG |
| system 8 | gyrAWT-R6-BT | CGACCAGGGCTGGGCCATGC |
|  | T200gA94AAL-4a | aagtgccgggtatctaacgtatGCGACGCGTCGATCaACA |
|  | T200gA94CAL-5g | aagtgccgggtatctaacgtatGCGACGCGTCGATgTACC |
|  | embBR16-1-BT | AGCGCCAGCAGGTTGTAATAC |
|  | T275eB306GTL-4a | acgtgacataatcgaaccctgcGACGGCTACATCCTGaGCG |
|  | T168eB306TTL-3t | gacggacgcatcggaaatcaaatGGCTACATCCTGGGCtTT |

**Microarray probe array**

| Probe | sequence |
| --- | --- |
| gbTAG197 | 5‘-NH2-（T15)-gtctccgcacgcaatgtttagt |
| gbTAG264 | 5‘-NH2-（T15)-attccgctgtagggttcgtatg |
| gbTAG200 | 5‘-NH2-（T15)-aagtgccgggtatctaacgtat |
| gbTAG275 | 5‘-NH2-（T15)-acgtgacataatcgaaccctgc |
| gbTAG168 | 5‘-NH2-（T15)-gacggacgcatcggaaatcaaa |
| gbTAG198 | 5‘-NH2-（T15)-aacgtgcggataagactcggaa |
| gbTAG206 | 5‘-NH2-（T15)-gactcgacaggacttcaaatgg |
| gbTAG104 | 5‘-NH2-（T15)-gtcgcgtaagattcggtcagtc |
| gbTAG221 | 5‘-NH2-（T15)-gtgagggcggtcgatacactat |
| gbTAG270 | 5‘-NH2-（T15)-actttcgcccgaactacaacag |
| gbTAG301 | 5‘-NH2-（T15)-aggtatcgccgcaaatctatcc |
| gbTAG202 | 5‘-NH2-（T15)-tctgctcaagtccgggttagtc |
| gbTAG235 | 5‘-NH2-（T15)-acgacactcacacggggtatta |
| gbTAG217 | 5‘-NH2-（T15)-cctccgatgtgccgttagactt |
| gbTAG279 | 5‘-NH2-（T15)-gacatttagttccgctcgctta |
| gbTAG205 | 5‘-NH2-（T15)-aggacggtttcttacgggatag |
| gbTAG171 | 5‘-NH2-（T15)-tatccctactcgttgcatgaca |
| gbTAG245 | 5‘-NH2-（T15)-ctgctcgttattagtgcttcgc |
| gbTAG257 | 5‘-NH2-（T15)-actccccgacacgagatatgag |
| gbTAG209 | 5‘-NH2-（T15)-accttacgttcgacactcctct |
| gbTAG108 | 5‘-NH2-（T15)-cagtcgccgtatagagcataga |
| gbTAG277 | 5‘-NH2-（T15)-gtccaacggtcacgctaatttt |
| gbTAG273 | 5‘-NH2-（T15)-tgccgcgaaatcacactttaca |
| gbTAG260 | 5‘-NH2-（T15)-gtttccctactattcgcgtgtt |
| gbTAG282 | 5‘-NH2-（T15)-caacgcttacgtccttacatcg |
