# Supplementary material for: A Chip for Detecting Tuberculosis Drug Resistance Based on Polymerase Chain Reaction (PCR)-Magnetic Bead Molecule Platform
Source: Front Microbiol. 2018 Sep 7;9:2106. doi: 10.3389/fmicb.2018.02106 (PMC6143819; doi:10.3389/fmicb.2018.02106)
Supplement: Supplementary file 2 [file Table_2.docx]

**Example illustrates**


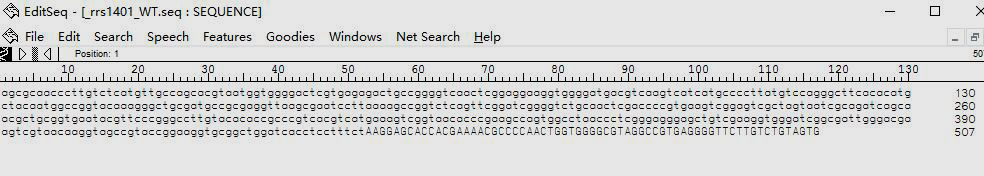


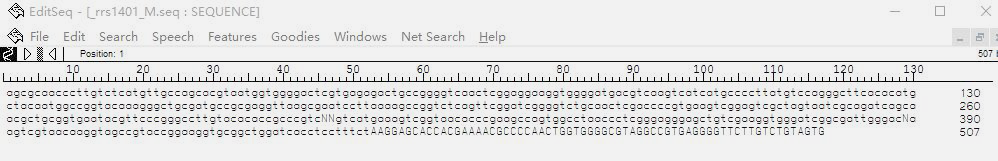


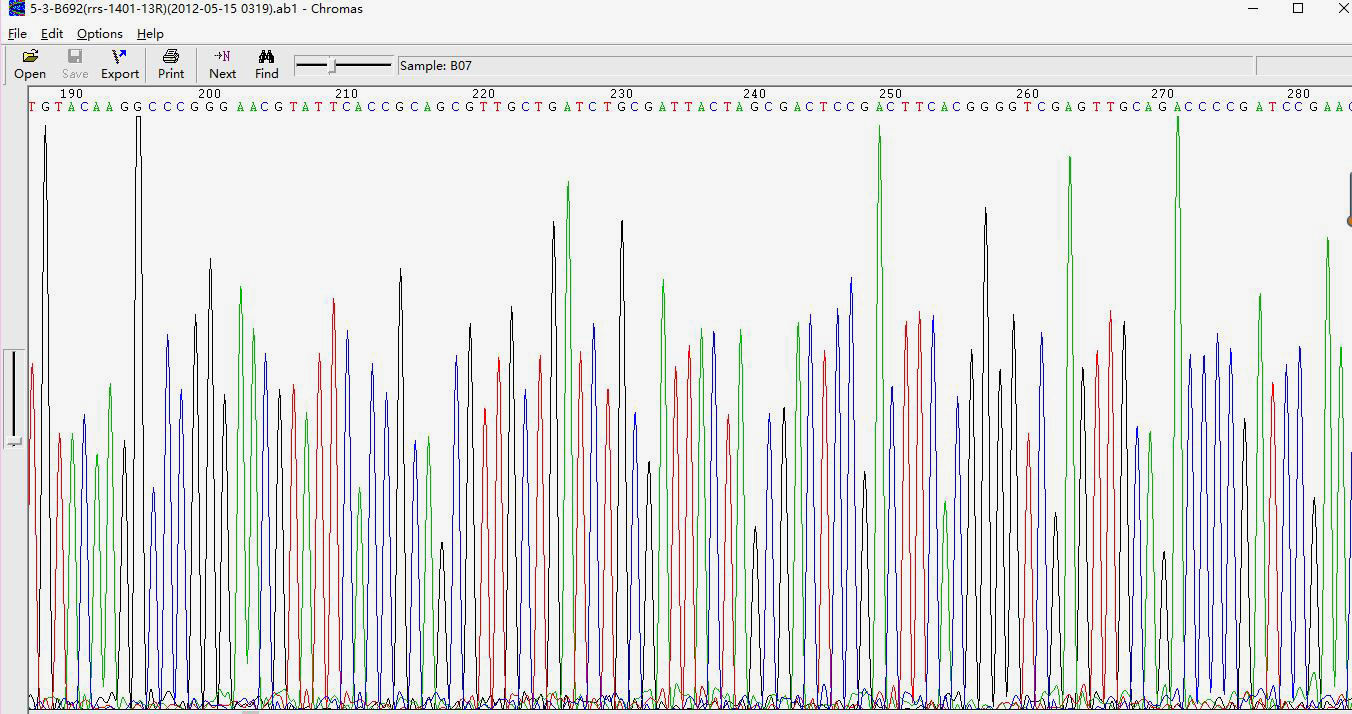


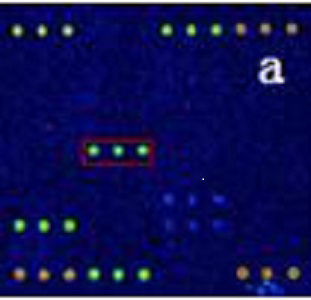


rrs1401(A→G): **According to supplement materials 2 (Microarray probe design, Microarray probe design description). The red box in the six column, and the six row are corresponding to Microarray probe design description, is shown as:** gbTAG217**, which corresponds to the** rrs1401 **Mutant probe in Microarray probe design.**
