# Supplementary material for: A Chip for Detecting Tuberculosis Drug Resistance Based on Polymerase Chain Reaction (PCR)-Magnetic Bead Molecule Platform
Source: Front Microbiol. 2018 Sep 7;9:2106. doi: 10.3389/fmicb.2018.02106 (PMC6143819; doi:10.3389/fmicb.2018.02106)
Supplement: Supplementary file 3 [file Table_3.docx]

**PCR reaction condition**

| **（℃）** | 37 | 96 | 94 | 72 | RAMP | 56℃ | 72℃ | 72 | 4 |
| --- | --- | --- | --- | --- | --- | --- | --- | --- | --- |
| **time（s）** | 600 | 600 | 25 | 1 | 0.2℃/s | 20s | 45s | 300 | --- |
| **Cycles** | 1 | 1 | 35 | | | | | 1 | 1 |
